# Public subsidies for professional football clubs: a conceptual political economy analysis

**DOI:** 10.3389/fspor.2026.1774307

**Published:** 2026-05-11

**Authors:** Johannes Baumeister, Joel Maxcy, Markus Kurscheidt

**Affiliations:** 1 BaySpo – Bayreuth Center of Sport Science, University of Bayreuth, Bayreuth, Germany; 2 LeBow College of Business, Drexel University, Philadelphia, PA, United States

**Keywords:** finance, geopolitics of sport, market power, monopoly, soccer, sport economics, sport governance

## Abstract

Despite the economic growth of professional football in Europe, clubs continue to receive substantial public subsidies, raising questions of legitimacy. While the relationship between public authorities and professional football has been studied from various disciplinary perspectives, an integrated theoretical framework remains absent. This conceptual study therefore adopts a political economy approach to systematize and integrate theories from sport economics and microeconomics, considering the political environment, aiming to setup a more coherent analytical model. For empirical and policy illustration, we draw specifically on the German Bundesliga, which exemplifies a balanced governance model combining corporate autonomy, democratic control by membership-based clubs, and public support. We advance two key propositions: First, the institutional relationship between clubs and local authorities can be conceptualized as a (local) bilateral monopoly with asymmetric market and bargaining power favoring the clubs. While local authorities hold a jurisdictional monopoly by law and through league restrictions on relocations, clubs may exert (economic) power due to effective barriers to entry for competing teams. Second, although market failures, externalities, and public-good characteristics can justify public support, subsidies may also serve political aims related to merit goods and distributional objectives. Public funding can thus either enhance overall welfare or sustain a monopoly operating at a loss. In conclusion, we argue for a strategic repositioning of the state as an active investor rather than a reactive financier. This approach may mitigate risks of government failure and prevent loss of control to investors with external – possibly geopolitical – interests.

## 1 Introduction

Professional football has become a global phenomenon, captivating audiences worldwide. Major national leagues and European competitions draw millions of viewers; the UEFA Champions League final alone reaches around 450 million spectators. While top clubs operate as global brands, the impressive total attendance of 221 million at European club matches in 2022/23, alongside a growing fan base in lower tiers, highlights clubs' deep connection with their local communities (1). This translates into substantial financial growth: revenues in Europe's top divisions have risen by 71% over the past decade, reaching €23.9 billion. Yet, financial strain – exacerbated by COVID-19 – has driven up debt, leaving 38% of top-division clubs with negative net equity by 2022 (2).

Despite lacking a coherent normative framework (3), professional football has always been entangled with politics (4). Economic expansion has prompted regulatory interventions, including competition policy and labor market regulation. Its significant societal impact has also led to various forms of direct and indirect support from public authorities, particularly at the local level. Key areas include infrastructure funding, assistance for financially struggling clubs – both often the subject of intense debate – and sponsorship activities by state-owned companies. These supports share a common trait: substantial political interest in maintaining a degree of opacity. Historically, as professional football has become increasingly commercialized, the relative share of public subsidies has declined, even though absolute subsidy amounts have continued to grow (5).

For this study, we use the term *public subsidies* broadly to cover all forms of government support – direct transfers, indirect benefits, public investments, or bailouts. While national and regional policies are acknowledged, the primary focus is on local-level subsidies due to their proximity to clubs and the distinct governance and bargaining dynamics involved.

Professional sport can serve as a platform for political and economic agendas (6). Yet, strategic planning is complicated by football's peculiarities: most European clubs are utility rather than profit maximizers (7), embedded in complex stakeholder structures – especially when a professional team remains legally tied to a democratic member-based association. These clubs compete in highly competitive leagues while maintaining local monopolies. Cultural and social factors further challenge purely economic explanations, evident in irrational investors (8) or the anomalies of football share markets (9). Consequently, research struggles to fully capture football's market dynamics and its ties with public authorities.

Scholarly debate has been shaped by studies on elite sport funding (10), mega-events (11), and U.S. team sports (12). Across these areas, the legitimacy of subsidies is frequently questioned and explained through new political economy approaches, highlighting politicians' motives, information asymmetries, and lobbying (13). Regarding professional football, the literature is advanced but fragmented, covering legal (8, 14), governance (15, 16), political (4), politico-economic (17, 18), economic (19), and sociological (20) perspectives. However, all these approaches are confronted with two major empirical and analytical challenges, which remain unresolved: first, why substantial public subsidies persist despite weak empirical evidence of long-term economic benefits; and second, why local authorities repeatedly appear to negotiate from a structurally weaker position despite holding formal regulatory authority. A more coherent approach toward an integration of existing theories is still lacking.

This conceptual study aims to systematize and connect these insights, bridging fragmented research. Using microeconomics as an integrative domain theory within a political economy approach (21), it addresses two research questions:

(1) How can the market and power relationship between professional football clubs and local authorities be conceptualized within established market-structure frameworks?
(2) How can subsidies for clubs be microeconomically justified to theoretically underpin current funding practices?

By drawing upon various theories from (sport) economics and political economy, the study aims to contribute to a more integrated conceptual understanding of the market and political relationship between public authorities and professional football clubs.

The paper proceeds as follows: the next chapter outlines our analytical approach, presenting an integrative theoretical model, and elaborates on three guiding theories in sport economics. Based on this framework, we discuss two key propositions addressing our research questions. Finally, we broaden the scope by adopting a macro perspective, integrating theories from the political environment to develop a broader understanding of actors' intentions and stakeholder relationships.

## 2 Analytical approach: an integrative theoretical model

Given the disciplinary fragmentation of the literature, this study makes a conceptual contribution. We aim to abstractly understand a situation or problem by identifying key patterns, connections, and underlying properties (22). Conceptual work can be demanding, as it lacks the structured research design of empirical studies (23). Yet, high-quality conceptual papers can strongly influence their fields by bridging theories, linking disciplines, offering multi-level insights, and broadening perspectives (24). In this context, our analytical approach is characterized by a process of reflexivity among the author team aiming at a coherent synthesis of individual theories.

### 2.1 Theory synthesis: domain and method theory

Our approach follows pertinent procedures of theory synthesis (23), which enables us to integrate diverse theories into a unified conceptual framework. In this sense, the notion “method theory” refers to an analytical lens rather than a research method in the empirical sense. We apply Lukka and Vinnari's (21) distinction between domain theory – the substantive field we aim to develop – and method theory – a meta-level conceptual system used to study it. The interplay between both shapes our understanding of the domain. Following MacInnis (22), we pursue two strategies: outlining propositional inventories within the domain theory and relating method theories to connect previously separate phenomena. This enables the development of broader, integrative perspectives.

### 2.2 Theoretical framework

**Figure 1:**
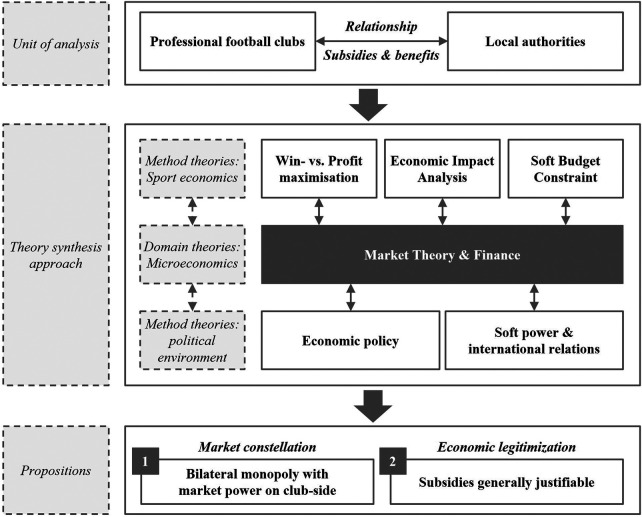
Theoretical framework.

Our theoretical framework, as depicted in Figure 1, begins with the unit of analysis: the relationship between professional football clubs and local authorities, with a focus on subsidies and benefits provided to the clubs. Our domain theories are grounded in microeconomics, drawing on market theory as the central analytical foundation and complemented by finance-related concepts. Market theory guides our understanding of competitive structures, pricing, and market power, while finance theory provides analytical tools for assessing investment under uncertainty, risk allocation, and the valuation of tangible and intangible benefits. For our theory synthesis, we integrate three method theories from sport economics – win vs. profit maximization, economic impact analysis, and the soft budget constraint – which we will detail below. While the microeconomic domain theories provide the core explanatory structure, these sport-economic approaches function as complementary analytical lenses that refine and contextualize our propositions regarding market structure and the economic justification for subsidies.

Proposition 1 conceptualizes the club-municipality relationship as a bilateral monopoly. Market-theoretic constructs such as entry barriers and bargaining power explain the structural setting, while finance concepts – notably sunk costs, asymmetric information, and risk allocation – clarify why bargaining outcomes may favor clubs. The sport-economic method theories illustrate how clubs' strategic objectives and financial constraints shape bargaining outcomes.

Proposition 2 examines the conditions under which subsidies can generate social and economic benefits. Here, market theory highlights externalities and welfare effects, whereas finance theory provides tools to assess intertemporal costs, risk-sharing, and the valuation of intangible benefits. Method theories demonstrate how clubs' incentives interact with social objectives.

Our analysis is based on the insightful example of the German Bundesliga, which is characterized by a balanced governance model of corporate autonomy, democratic control by membership clubs, and public support.

In the discussion, we extend the framework beyond microeconomics by briefly incorporating political and geopolitical perspectives as a broader interpretive extension toward economic policy and international relations. This allows us to link market-structure analysis with broader strategic considerations regarding state involvement. This layered approach ensures that the analytical focus remains microeconomic, while still enabling a broader macro-level reflection on policy, governance, and international dynamics.

### 2.3 Method theories from sport economics

As outlined above, the following three method theories from sport economics complement the microeconomic domain framework: win vs. profit maximization, economic impact analysis, and the soft budget constraint. These theories do not replace the core microeconomic perspective but specify how professional football clubs behave, how their economic effects are assessed, and how financial constraints operate in practice. They therefore provide the behavioral and institutional mechanisms that underpin Propositions 1 and 2.

#### 2.3.1 Win vs. profit maximization

The objectives of professional sport teams have been debated for over 50 years. Sloane (7) argued that profit maximization – the standard goal in most industries – was ill-suited to football, characterizing clubs instead as utility maximizers, often interpreted as win maximizers operating under budget constraints (25). A widely accepted distinction holds that American closed leagues are profit-driven, while European open leagues with promotion and relegation follow win-maximizing logics (26).

In football, there is a strong link between team success and player expenditure, with revenues and budget constraints determining spending (27). Because relative team quality matters most, win maximization can drive clubs into arms races, often leading to overinvestment and financial instability. Pure win maximizers lack sufficient buffers or risk management, increasing financial vulnerability (28).

Despite numerous studies, empirical evidence on profit vs. win maximization remains inconclusive (26). A likely reason is that clubs pursue broader utility functions that include more than just sporting success. Madden (29), for example, introduces fan welfare as an objective. Social and ecological goals also increasingly influence club strategies (30, 31).

Club objectives are predominantly interdependent, combining complementary and competing goals. Priorities vary between clubs and may change over time (31). Losses can be part of an intertemporal budget strategy: in markets with growing revenues, win-maximizing clubs may ultimately generate higher profits than profit-oriented rivals (27, 32).

To address this complexity, Prinz and Thiem (33) propose value maximization as a long-term goal. This aligns well with the 50 + 1 rule in the German Bundesliga^1^, as club members, who serve as the ultimate decision-making body, have a vested interest in their club's long-term stability and growth.^2^ This structure also ensures profits are retained for risk buffers or reinvestment in sporting success.

In conclusion, we interpret German professional football clubs as value maximizers, combining sporting and social objectives under a dynamic budget constraint. This reflects their public-good character, which we explore further in our analysis. Within our framework, this value-maximizing behavior has two important implications. First, it helps explain why clubs prioritize sporting relevance and long-term positioning over short-term profitability. As a result, clubs may be more willing to accept financial risks and push for favorable conditions in negotiations with local authorities, thereby potentially strengthening their bargaining position within a bilateral monopoly setting (Proposition 1). Second, by generating sporting success, identity, and social cohesion, clubs produce positive externalities that form part of the microeconomic justification for public support (Proposition 2).

#### 2.3.2 Economic impact analysis (EIA)

Public subsidies for professional sports teams are often justified by referencing their supposed economic benefits, typically based on Economic Impact Analyses (EIA). These *ex-ante* studies, however, tend to be exaggerated, politically motivated, and methodologically flawed. Key issues include confusing gross with net benefits, unclear impact boundaries, and inflated multipliers for indirect and induced effects (34). By contrast, a robust body of *ex-post* research finds little evidence that professional teams or new facilities generate meaningful economic growth (12). Reasons include the small economic scale of teams, leakage effects due to nonlocal spending, and substitution in consumer behavior (35).

While most studies focus on U.S. franchises, findings generally apply to Europe (36). Results are more ambiguous for urban districts (37) and peripheral regions (19), where some positive tangible effects are observed.

To capture the total economic value of professional sport, intangible effects such as externalities and public goods must be included (38). Valuing these intangibles is challenging, as they are difficult to quantify and operationalize. Methods include analyzing substitution markets (e.g., changes in property values around sport facilities) or using hypothetical markets such as contingent valuation to estimate willingness to pay for an event, a facility, or a local club. However, these approaches are prone to various biases that limit accuracy (38, 39).

Intangible benefits can be grouped into socialization and representation effects (Table 1). *Socialization effects* are inward-facing and include improved quality of life, subjective well-being, happiness, and life satisfaction (40, 41). They may also foster regional identity and collective memory (20, 42), supported by constructs such as basking in reflected glory and civic pride (43, 44). Professional sport may also enhance social capital (45).

**Table 1:** Potential economic impact of professional sport.

Tangible effects (operationalizable via official statistics and accounting data)	Intangible effects (operationalizable primarily through indirect indicators or primary data collection, e.g., surveys, social research)	
Economic growth effects	Socialisation effects (inwards)	Representation effects (outwards)
- Value added - Employment - Income level - Tax revenue	- Leisure and entertainment - Identification and social cohesion	- Location marketing for attracting direct investments, influx of productive employees and students, tourists

Contingent valuation studies often assess the existing value of sports clubs, which reflects many of these socialization effects. They consistently demonstrate residents' general willingness to pay for the continued existence of their local team (46, 47).

In times of globalization, *representation effects* have gained increasing importance. Event signaling has become a valuable tool for showcasing a location's quality to attract new businesses, residents, or tourists (48). Professional football clubs serve as significant image builders for cities or regions, influencing their location brand (17, 49). Stadiums may even become iconic landmarks (43, 50). In particular, in cities with a post-industrial past, football can play an important role in reshaping external perceptions and supporting urban regeneration narratives (51). The value of this brand-building depends on success, media attention, and location size. While in big cities clubs are one of many image factors, in smaller towns they may become defining landmarks (49).

Many EIA studies highlight benefits but overlook negative externalities, such as increased traffic, security concerns, or environmental impacts. Including these is essential for an accurate valuation (12, 38).

In conclusion, the existing literature provides no evidence of significant tangible economic benefits from professional football clubs. While socialization and representation effects are challenging to quantify, they are verifiable and contribute to the overall value of professional sport. Within our framework, EIA primarily informs Proposition 2 by structuring the assessment of externalities and public-good characteristics. Whether these effects justify public subsidies depends on the specific context, subsidy size, and further research into intangible effects.

#### 2.3.3 Soft budget constraint (SBC)

Despite the growing financial stability of major European leagues, smaller leagues and lower-tier clubs still face significant financial challenges (52). The COVID-19 pandemic worsened this situation: between 2020 and 2022, European first-division clubs reported combined losses of €11 billion, and 38% had negative net equity by 2022 (2). Nevertheless, clubs demonstrated remarkable resilience: from 2020 to 2023, across Europe's top two tiers, only 11 clubs per year declared insolvency out of approximately 1,500 clubs (2).

This paradox of persistent deficits alongside few bankruptcies is explained by the soft budget constraint (SBC). Introduced by János Kornai in the 1980s, the SBC describes organizations that expect bailouts in financial distress, unlike capitalist firms that must match revenues and costs to avoid bankruptcy. Kornai distinguishes a continuum from hard to soft budget constraints: bailouts can occur either ex ante to prevent crises or ex post to resolve them (53).

Storm and Nielsen (52) applied the SBC concept to football, showing that clubs with chronic deficits are often rescued by wealthy owners, sponsors, or public funds. Although bailouts are not guaranteed, their high probability shapes club behavior. Some clubs are “too big to fail” because of their social and cultural role.

The SBC can manifest in various forms:

- Soft prices: below-market stadium rents, public sponsorships, etc.
- Soft taxes: tax exemptions, favorable tax arrangements, etc.
- Soft subsidies: public or private financial support.
- Soft credits: debt guarantees, postponed repayments, etc.
- Soft investments: infrastructure financing, favorable ownership structures, etc.
- Soft accounting: accounting manipulation and lack of transparency (54).

Recent research provides empirical evidence of SBCs in areas such as stadium financing (55), lower-tier football (56), non-professional football (57), and even insolvent clubs (58).

Andreff (59) identifies determinants of SBCs at three levels:

- Micro-entrepreneurial: win-maximizing behavior, fan pressure for talent acquisition, poor governance
- Meso-industrial: promotion–relegation system, talent arms race
- Macro-national: tax policies, fiscal discipline

Regulatory approaches can mitigate SBCs, such as UEFA's Financial Fair Play (FFP) rules within sport governance or the European Union's state aid regulations at the political level (58, 60). SBCs have clear consequences: they foster inefficiencies due to limited pressure for sound financial management (54), encourage overinvestment and risky spending (60), and create competitive distortions by pressuring financially disciplined clubs to adopt similar practices (61). Within our framework, SBC primarily reinforces Proposition 1 by explaining altered risk incentives and the expectation of external support. Anticipated bailouts may weaken financial discipline and increase clubs' willingness to engage in aggressive bargaining with public authorities. While public actors may gain leverage when support is conditional (62), political and reputational pressures often limit their ability to credibly threaten non-intervention. With regard to Proposition 2, it highlights potential financial vulnerabilities that may trigger public intervention, without in itself constituting a normative justification for subsidies.

## 3 Proposition 1: clubs and local authorities as a bilateral monopoly with market power on club's side

Classical microeconomic models assume competitive markets with many actors on both the supply and demand sides, each with a small market share. However, this often does not reflect reality. Here, we demonstrate that the relationship between a city and a club can be viewed as a bilateral monopoly. Furthermore, we argue that market power in this setting lies primarily with the club.

Monopolies can arise due to regulatory or market-induced barriers to competition. Clubs seek infrastructure and financial support to maximize sporting and social value, while local authorities aim to secure rental income and economic spillovers. On both sides, there is typically a single supplier and a single consumer, resulting in a bilateral monopoly.

### 3.1 Local authority as a monopoly: barriers to relocation

In contrast to American sports franchises that can relocate, German (and most European) clubs face regulatory and market-induced barriers that make relocation nearly impossible. Regulatory barriers stem from the licensing regulations of the German Football League (DFL), which governs the first and second divisions in Germany.^3^ A club's license is non-transferable (§1 Nr. 1) and requires a stadium in the club's registered location (§6 Nr. 1). Corporations must be based at the club's registered office (§4 Nr. 1) and remain under majority control of the member association (DFL Constitution §8 Nr. 3).

Market barriers reinforce this immobility. Even if relocation were legally possible, members – who, under the 50 + 1 rule, hold ultimate decision-making power – are deeply rooted in the home city and would most likely oppose a move. Hence, only neighboring counties – often economically weaker – or, to some extent, higher administrative units could replace the city as a partner. However, both alternatives are largely theoretical and do not significantly challenge the local authority's monopoly status.

While these regulatory and market barriers are specific to the German context, similar mechanisms – including licensing requirements, membership control, and entrenched local fan bases – exist across most European professional football leagues. In practice, club relocation is extremely rare, highlighting the general relevance of the local authority's monopoly position beyond Germany (e.g., Wimbledon FC in England being a rare exception).

### 3.2 Local club as a monopoly: barriers to enter the football market

Unlike U.S. major leagues, European football lacks explicit monopoly regulation (63). Yet, various regulatory barriers hinder new competitors. Clubs cannot freely choose their location, licenses are non-transferable, and in Germany, a club must be a German Football Association (DFB) member for at least three years to get a license (DFL Licensing Regulations, §1 Nr. 2).

Entry into the professional leagues requires sporting success: new clubs must start in amateur divisions and climb the pyramid, a process taking years and substantial funding. Moreover, the number of professional spots is capped at 56.^4^ Even if an investor were willing to take on this long and costly path, they would typically reject the 50 + 1 rule, which ensures club member control over any corporation.^5^ Theoretically, new competitors could attempt to enter the football market by rejecting these regulatory barriers. However, this would require establishing an entirely new league system capable of competing with the entrenched monopoly structures of national and international leagues and associations – effectively challenging the entire system.^6^

Professional football clubs exhibit many characteristics of a natural monopoly. High fixed costs for stadium infrastructure and substantial committed expenditures on player salaries, combined with low marginal costs per consumer – mathematically characterized by the principle of subadditivity – create a significant market-induced barrier. Existing clubs benefit from their first-mover advantage, which creates economies of scale through high brand value and strong fan loyalty, resulting in low price elasticity. These factors, combined with strong network effects in fan communities, discourage new market entry. Market reach is mostly local or regional; only a few top clubs succeed as global brands (5). Under these conditions, new competitors would require substantial capital while facing limited revenue potential in an already shared market. Such investments are highly risky, as specific infrastructure investments often constitute sunk costs in case of failure (64).

It is worth noting that some major European and German cities have more than one professional club, demonstrating the limits of natural monopolies. However, this typically occurs in large markets where the revenue potential is sufficient to sustain more than one club despite high fixed costs. Most of these additional clubs historically developed by catering to distinct target groups – whether based on locality, social class, political affiliation, or ethnicity (e.g., Atlético Madrid, Celtic Glasgow, Inter Milan, FC St. Pauli Hamburg). Successful secondary clubs establish a strong brand value and a loyal fan base, often fueled by intense rivalries with local competitors or reaching out to regional or national markets by their strong positioning.

Outside large metropolitan areas, there is usually just one dominant professional club. Beyond competition from other football clubs, substitutes such as other professional sports teams and the broader local entertainment industry may challenge a football club's monopoly status. However, in practice, these alternatives generally remain imperfect substitutes. The cultural significance of professional football, its deep-rooted relevance across all social classes, and most clubs' loyal and sizable fan bases make it uniquely dominant. Once a football club establishes its presence, other sports rarely match its local economic, social, or symbolic impact. For example, even in Germany, with strong handball, basketball or ice-hockey leagues still third-division football generates higher attendance and revenue than these alternative sports. Similar patterns can be observed in France, Spain, and Italy, where second-division football clubs largely outperform local handball, basketball, or rugby teams in terms of spectator numbers, media coverage, and sponsorship.

In summary, both regulatory and market barriers protect local clubs' monopoly status. Only in a few large cities do oligopolistic structures emerge, but even then, there is often a clear market leader acting like a monopolist.^7^

### 3.3 Football market's specific risks for investments and subsidies

Professional football poses specific challenges that complicate private investment. Although economic strength and sporting success correlate long-term, football remains inherently uncertain, with frequent unexpected results (65). Negative events like relegation or missing international competitions can create path dependencies that jeopardize prior investments. On-field failure usually reduces revenue potential significantly; for instance, average revenue in the Bundesliga drops from €247.4 million to €43.7 million after relegation (66). Clubs then often need stadium rent adjustments or additional funding, and in the worst-case face insolvency or denial of a license.

The 50 + 1 rule ensures member-majority control, limiting investors' influence. Investors depend on club members for decisions affecting the success of their investment and potential dividend payouts. Internally, decision-making often resembles politics more than corporate governance, with high public involvement. Moreover, the legal structure of a member-based association limits personal liability and financial disclosure, encouraging a short-term focus on winning over long-term stability and promoting overinvestment and risk-taking.

Given these circumstances, private operators are often reluctant to fully finance projects and seek public co-funding instead. This pressures local authorities to support clubs. High public spending elsewhere fuels competition between cities, creating an “arms race” for favorable conditions. Although EU state aid rules generally restrict subsidies, difficult conditions for private investors often make public support both feasible and necessary (8). Ultimately, the financial risk lies mainly with the public sector.

### 3.4 Market power: why clubs hold the advantage

In the bilateral monopoly between professional football clubs and local authorities, market power is clearly tilted in favor of the clubs. One reason is information asymmetry. Clubs have greater market expertise, creating agency problems (67). Public authorities often lack full market information, strengthening clubs' bargaining power. Furthermore, clubs can exploit hidden intentions, causing hold-up issues like overinvestment followed by stadium rent disputes.

As discussed under soft budget constraints, many clubs – particularly those in cities with a single dominant club or in the top leagues – seem to be “too big to fail”. Their social and cultural significance creates intense political pressure on local authorities to provide financial support (68). This, in turn, softens the budget constraints clubs face and enhances their market power. Club officials, aware of their fan base's influence, sometimes escalate conflicts to amplify this pressure. Political incentives reinforce this: public choice theory suggests politicians act in self-interest, and supporting local clubs can yield electoral benefits. Strong ties between clubs and local politics enable lobbying, while successful teams boost politicians' popularity by allowing politicians to “bask in reflected glory” (43).

Managerial factors further weaken public authorities' position. Investments in highly specific infrastructure, like stadiums, risk becoming sunk costs. Subsidies or lenient terms may be preferable to leaving a stadium empty – the classic “white elephant” problem. A journalist aptly described Lech Poznań's case: “Lech cannot afford to pay a high rent, the city cannot afford to have an empty stadium, [and] both sides are somehow fated to stay with each other” (11).

Regulations on insolvency have mixed effects. Before 2007, insolvency meant forced relegation; now, it leads only to a nine-point deduction (DFL licensing regulations §11 Nr. 5) – even this was suspended during COVID-19. This reduces the pressure on local authorities to bail out struggling clubs, since insolvency no longer necessarily threatens a club's professional status. Yet lenient rules also encourage mismanagement, with some clubs using insolvency strategically (69). Since public authorities are often key creditors, insolvency can cause significant financial losses.

In conclusion, as previously established, the existence of soft budget constraints – combined with information asymmetries, political incentives, and sunk costs – creates a structural imbalance of market power, favoring clubs over local authorities.

### 3.5 Consequences of bilateral monopoly and club's market power for price determination and market behavior

In a bilateral monopoly, pricing is not determined by traditional supply and demand curves. Instead, the outcome depends on the bargaining process between buyer and seller. An agreement is reached if the buyer's maximum willingness to pay exceeds the seller's minimum acceptable price. The final price falls between these two extremes and is influenced by the relative bargaining power of each party (70).

Price formation ties directly to expected benefits, which creates challenges for local authorities. While stadium rents can be calculated using market principles, subsidy components depend on the club's (socio-)economic impact. As discussed, quantifying intangible benefits like social effects is difficult and often contested. Clubs and authorities have incentives to overstate these benefits – clubs to strengthen their bargaining position, authorities to justify subsidies.

Both sides are motivated to reach an agreement due to limited alternatives. But with market power mainly on the club's side, outcomes usually favor the club. This discourages clubs from seeking other funding and increases their willingness to take financial risks. Ultimately, public authorities are in a structurally weaker position, leading to favorable conditions for clubs at the taxpayer's expense.

However, some factors can weaken clubs' power and strengthen public bargaining positions. Financially well-equipped clubs – particularly those backed by a wealthy sponsor or patron – are in a weaker position to demand public support. Local substitutes can also reduce a club's leverage – for example, public benefits from professional football in Munich could shift to Bayern Munich instead of 1860 Munich. While local authorities retain their monopoly status, clubs cannot easily relocate and must compete for funding. Overall, the bilateral monopoly logic is strongest for clubs that rely on local public funding, typically outside the top financial tier or in cities with a single dominant club. In cities with multiple professional clubs, the model primarily applies to the dominant club, while follower clubs typically hold weaker bargaining positions. More generally, where clubs have substantial private backing or face local competition, bargaining power may shift, and the structural advantage of the club is reduced. Other mitigating factors include weaker fan leverage or significant alternative uses of infrastructure. Nevertheless, political, reputational, and social pressures can still shape negotiations, even if the structural dominance implied by a bilateral monopoly is less pronounced.

## 4 Proposition 2: subsidies for professional football clubs economically or politically justifiable

Public support for professional sports teams is often criticized in academic literature (12, 36). However, there is a valid basis for subsidies from a regulatory perspective. Here, we examine when and why state intervention can be justified, rather than debating the exact scope of public measures.

We first address the economic rationale, focusing on market failures that justify public intervention, then the political perspective, and finally apply this to two cases: welfare-enhancing subsidies and support for failing monopolies.

### 4.1 Market failure: external effects and public-good characteristics

As discussed in our section on economic impact analysis, professional football generates significant intangible benefits. Since these socialization and representation effects are not captured by a market-based price mechanism, they create market failure: private marginal utility is lower than social marginal utility, leading to prices that are too low and an output level that is suboptimal. In financial theory, this phenomenon is referred to as an external effect. Subsidies can address market failure by internalizing these positive externalities (71, 72).

Beyond external effects, many benefits have public good characteristics (46). Public goods are defined by non-excludability and non-rivalry. While stadium entry and broadcasts can be made exclusive, the sense of community, pride after a win, or improved city image cannot be restricted to paying fans. These effects are thus non-excludable.

Non-rivalry means one person's benefit does not diminish another's. For stadium seats, rivalry exists in a narrow sense, as a specific seat can only be occupied by one person. In a broader sense, rivalry only arises when capacity is reached. In economic terms, this is known as a club good. Moreover, shared experiences among fans can actually enhance consumption quality, a phenomenon described as a mob good (35). Broadcasts are non-rivalrous by nature, and in public interactions, significant mob good characteristics emerge. The more people engage with the club and interact as a group, the greater their personal fulfillment. Also, enjoyment typically grows with experience, making football clubs a connoisseur good (73).

Thus, we can conclude that a professional football club is a joint good with public-good characteristics. Haas (74) differentiates between the entertainment value and the result value of a football game. The entertainment value – determined by sporting quality, excitement, and service quality – is predominantly a private good, as it involves production costs and can be made exclusive. In contrast, the match result value can be classified as a public good, as it entails no rivalry and no excludability; individuals do not need to attend or even watch a game to experience pride or joy. On a societal level, football clubs foster communication, identification, and representation, reinforcing their public good nature.

More broadly, the very existence of a football club can constitute a public good (75). Applying the Total Economic Value (TEV) framework (76) illustrates that sports clubs create both use and non-use values: option value (the benefit of keeping the possibility to engage in the future), existence value (satisfaction from knowing the club exists), and bequest value (preserving the club for future generations). Given the socialization and representation effects of professional football clubs, these non-use values align with public-good characteristics: non-excludability and non-rivalry, which facilitate free-riding. Contingent valuation methods (CVM) provide an approach for potential empirical estimation of the TEV.

Stadiums themselves can exhibit public-good elements too. Landmark architecture and collective memories contribute to place branding and local identity, generating city-wide benefits beyond ticket holders (11, 43).

The public-good characteristics of professional football clubs can, from an economic perspective, be treated similarly to external effects (64). Public subsidies therefore serve as a mechanism to internalize these externalities and compensate clubs for the free-ride consumption of their social and cultural benefits by cities and their residents (12). This justifies public intervention in principle – though not necessarily in scope. As Standen (77) asserts: “Like any public good, the lack of a public subsidy would cause the good to be under-produced”.

From a legal perspective, German subsidy regulation requires that funding serves a common interest – which includes externalities and public good elements – and that no better alternative exists (78). As discussed in our first proposition, the monopoly-like nature of local professional football clubs means that no close substitutes are available. Moreover, Bundesliga status functions as a positional good (79): with a fixed number of teams, cities compete for the reputational benefits of hosting a club, creating positive externalities for host cities and negative ones for those excluded.

### 4.2 Political objectives: merit goods and distributional goals

Beyond resource allocation and market failure, professional football also raises socio-political considerations that pure economics alone cannot fully capture. The concepts of merit goods and distribution policy help to frame these political objectives.

The idea of merit goods, introduced by Musgrave in the 1950s, remains debated (71). The key point is that some goods should be promoted because they generate positive effects for individuals or society, even if market demand underestimates their true welfare potential. This gap often stems from imperfect information, non-rational behavior, or moral and ethical preferences. State intervention via regulation or subsidies thus reflects a political willingness to correct so-called defective preferences (80).

Some merit aspects of professional football – like socialization and representation – overlap with external effects and thus support market-based justifications for subsidies. Other claimed effects, such as better public health through promoting active sport or value transmission via fair play, lack robust empirical backing (81). This section focuses on the cultural dimension: cultural goods often receive public support because they are seen as beneficial beyond what markets would supply. As discussed in the section on soft budget constraints, football's cultural significance reinforces its political legitimacy for public funding. Even where direct externalities are limited, policymakers may seek to “correct” perceived defective preferences by subsidizing local clubs to ensure the continued provision of professional football as a cultural good.

Distribution policies pursue a fairer allocation of resources and reflect political choices beyond market logic as well. In German football, this appears in various media and governance rules:

- the legal obligation to show major sports events on free-to-air TV,
- public broadcasters buying Bundesliga rights,
- the antitrust approval of the league's centralized TV marketing.

Even the political support for the 50 + 1 rule can be considered a form of distribution policy, as it aims to prevent an excessive concentration of ownership and financial power among wealthy corporations or individuals.

In summary, while external effects and public good aspects justify subsidies on economic grounds, football's merit-good status and its role in fair resource distribution strengthen the political rationale. In the following, we analyze two specific constellations from an economic perspective: subsidies to raise public welfare and subsidies to support a monopoly at a loss.

### 4.3 Public subsidies to raise public welfare

As discussed, professional football generates external benefits that clubs cannot fully capture, leading them to operate below the socially optimal output level. This underproduction of external benefits creates an inefficiency that can justify public subsidies.

To illustrate this, we adapt a model developed by Fort (13) for U.S. major leagues to the European context.

**Figure 2:**
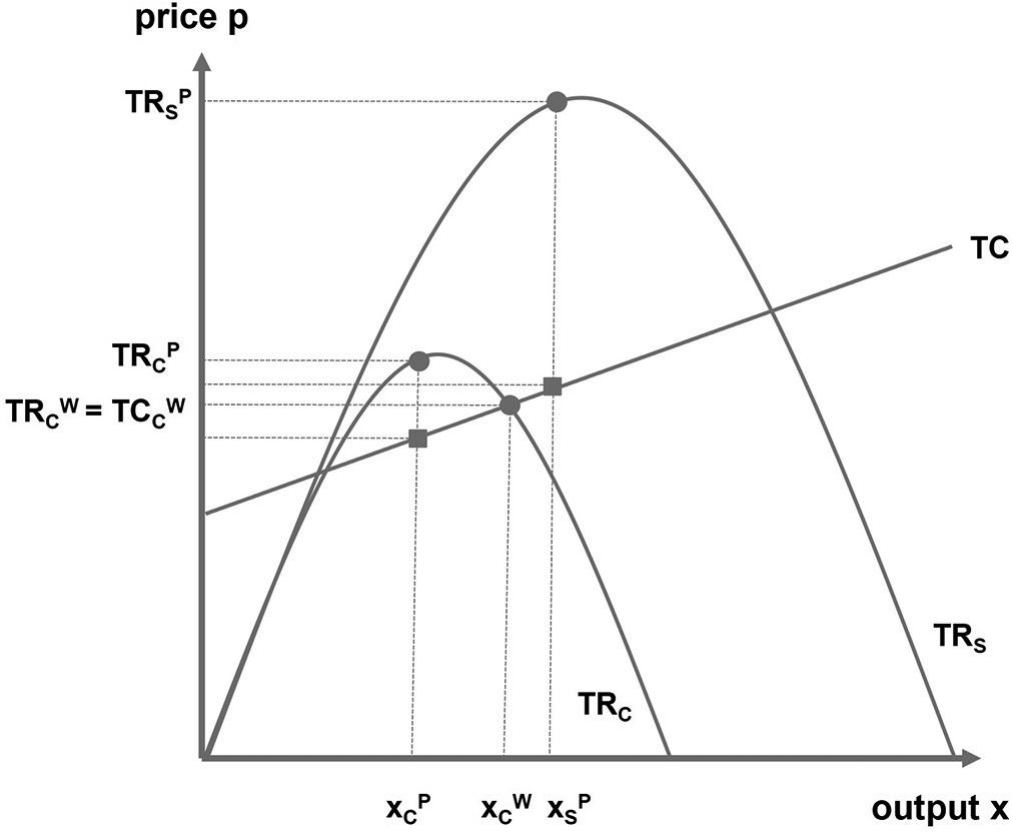
Public subsidies to raise public welfare.

 Figure 2 depicts a club's total revenue function TR_C_ alongside its total cost function TC. Assuming that greater investment in team quality directly improves sporting success (“money shoots goals”), a profit-maximizing club chooses the output level x_C_^P^, where the difference between private total revenue TR_C_^P^ and total costs is maximized. However, the full social value generated by the club – represented by the social revenue function TR_S_ – is higher, so the socially optimal investment level is x_S_^P^, where social total revenue TR_S_^P^ is maximized.

In North American leagues, bridging the gap between x_C_^P^ and x_S_^P^ usually requires public subsidies. In European football, however, the competitive structure means clubs often prioritize sporting success over profit maximization. As discussed earlier, most clubs operate at a level where total club revenues equal total costs – equivalent to win maximization under a budget constraint. In Figure 2, this corresponds to the win-maximizing point x_C_^W^, where the revenue function intersects the cost function (TR_C_^W^ = TC_C_^W^).

Therefore, win-maximizing clubs already generate more social welfare than profit-maximizing clubs, as all revenues are reinvested into team quality. In contrast, profits retained by club owners indicate a suboptimal allocation from a social perspective.^8^ Nevertheless, even win-maximizing clubs do not fully reach the social optimum. By subsidizing them, public authorities can raise the output level from x_C_^W^ to x_S_^P^, thereby further enhancing public welfare.^9^

Empirical studies support this logic: successful teams boost identification effects, improve public mood and self-esteem, and increase local consumer spending (82). A practical example is subsidizing a club's squad to secure promotion to a higher league, which raises club revenue and delivers broader social benefits.

### 4.4 Public subsidies to support a monopoly at a loss

**Figure 3:**
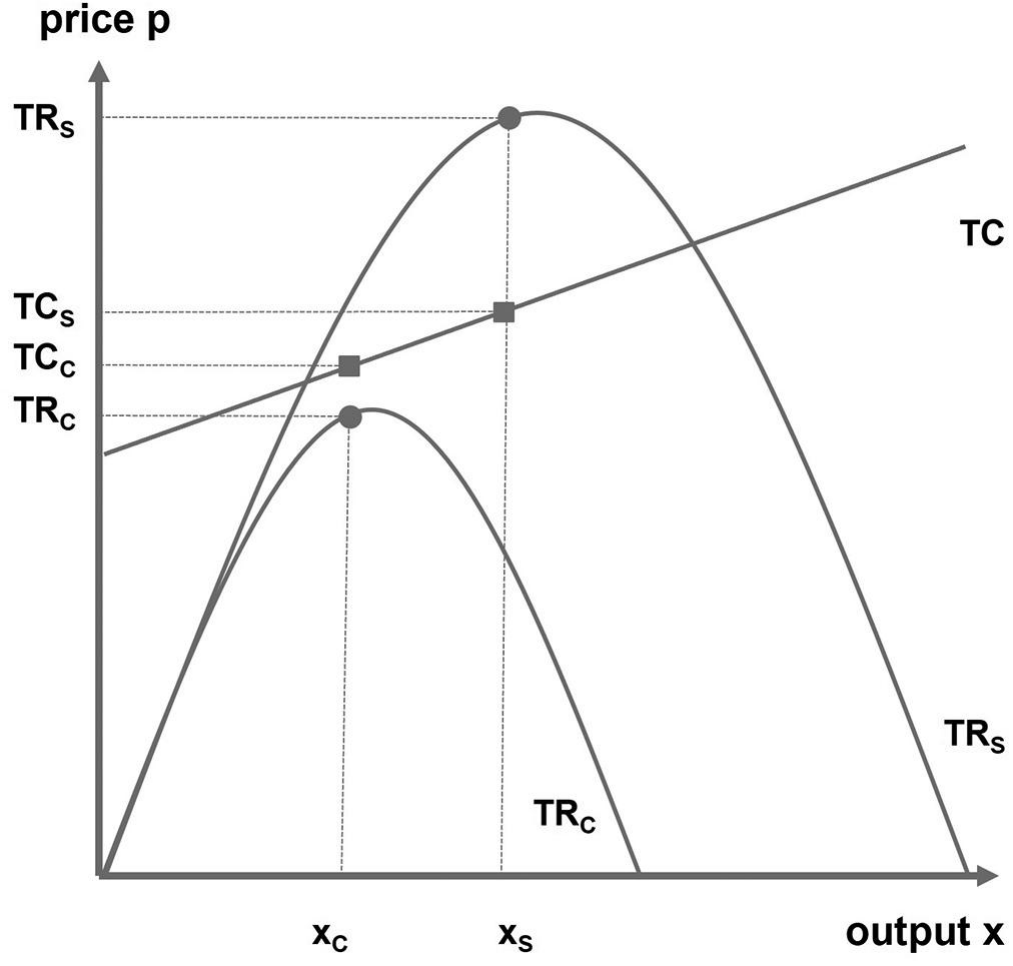
Backing a losing monopoly.

In some cases, the question arises whether professional football can be sustained at all without public subsidies – especially when financial support is needed to keep a loss-making monopoly alive (13). Figure 3 illustrates this scenario: the club's total cost function TC_C_ remains consistently above its total revenue function TR_C_. Without subsidies, the club would either go bankrupt or, if fixed costs allow, be forced to scale down its operations and withdraw from professional football to grassroots level.

In North American major leagues, teams in such a situation often relocate to more promising markets. However, as outlined in Proposition 1 regarding the bilateral monopoly between clubs and local authorities, this option is largely unavailable in European football. If no adequate substitutes exist for the local authority, the only way to achieve a positive socioeconomic outcome is through a subsidy covering the gap between club costs and revenues at output level x_C_. Since no profit is generated in this scenario, considerations of profit or win maximization are irrelevant.

Public support is generally justifiable if the total socioeconomic benefit outweighs the required subsidy. Figure 3 represents the case where a minimum subsidy ensures the continued existence of the professional football club. If the authority wants not only to preserve the club but also to maximize social benefits by improving sporting quality, the subsidy must be increased to push output up to x_S_ – paralleling the argument in Figure 2.

A practical example is a club in the third division, located in an economically weak region with low market potential. Here, subsidies can help balance competitive disadvantages, allowing the club to remain viable against financially stronger competitors.

## 5 Discussion and conclusion

Within the framework of our two propositions, we have examined the relationship between the state and professional football from a market and competition perspective. Proposition 1 highlights that professional football clubs often hold substantial market power within a bilateral monopoly with local authorities, shaping bargaining outcomes and influencing the terms of public support. Proposition 2 suggests that clubs can generate significant positive externalities and public-good effects that are not fully captured by market mechanisms, providing a potential economic and social rationale for state involvement. These insights show that purely microeconomic considerations are not sufficient for designing effective policies. They also provide the foundation for the subsequent discussion: in Section 5.2, we translate these structural and welfare-related findings into a strategic approach to public engagement, showing how local authorities can proactively shape club outcomes for broader urban, social, and economic benefits. In Section 5.3, we extend the perspective to the international and geopolitical dimension, illustrating how professional football interacts with soft power, nation branding, and cultural diplomacy.

### 5.1 Microeconomics are not enough

External effects, public-good characteristics, merit goods, and distributional goals provide a sound basis for justifying subsidies. Additionally, the bilateral monopoly structure, where clubs hold market power, creates competitive distortions and increases pressure on government action. However, these conditions alone do not automatically warrant intervention: a subsidy must also be advantageous – the benefits must clearly outweigh the costs – and preferable to alternative uses of public funds. From a regulatory perspective, interventions must align with policy objectives, conform to the existing system, and remain proportionate (71). Since these criteria are often ambiguous, measures to correct market failures can lead to government failure, causing misallocation and welfare losses (64).

Importantly, a distinction must be made between what can be justified *in principle* and what is likely to be welfare-enhancing *in practice*. From a microeconomic perspective, subsidies may be justified if positive externalities exceed costs, if market power leads to inefficient outcomes, or if social returns surpass private incentives. However, such theoretical conditions do not automatically translate into net public benefits in real-world settings. Policymakers must therefore evaluate opportunity costs, the distribution of benefits, potential rent capture by clubs, intertemporal fiscal implications, and the time horizon over which social returns materialize. Without such scrutiny, even theoretically sensible interventions may result in inefficient allocation or reinforce existing power asymmetries. A related concern is that a share of public support may ultimately be capitalized into higher player salaries rather than broader local economic benefits. Since professional athletes typically belong to high-income groups with comparatively low marginal propensities to consume locally, this may limit the multiplier effects of public spending and raises important distributional and efficiency concerns.

Furthermore, while our analysis focuses on local-level impacts, we acknowledge that subsidies at the national level can create redistributional effects between cities and clubs. Public support for one club may improve its competitive performance while reducing the relative position of others, potentially creating a zero-sum effect in terms of league outcomes. Nevertheless, local subsidies may still generate net social value through urban development, identity formation, and community cohesion, which are not captured by relative league performance alone.

While market failure theory provides a conceptual justification for subsidies, empirical validation remains crucial. Policymakers must assess not only whether positive externalities exist, but also their magnitude relative to the size of the subsidy and its opportunity costs. This includes evaluating potential crowding-out effects, “anyway effects” (benefits that would have occurred without public support), and the distribution of gains between clubs, local communities, and private investors. An effective evaluation framework therefore requires comparing the subsidy volume with measurable and intangible returns, identifying who ultimately captures rents, and considering the relevant time horizon over which benefits and fiscal risks materialize. Only if expected net social returns exceed costs under realistic assumptions can intervention be considered welfare-enhancing in practice.

Regarding subsidies for professional football, public authorities face key dilemmas: Is the goal to keep a monopoly alive or to maximize welfare? Should policy be short-term crisis response or long-term development? Should it react to external shocks or follow a strategic plan? Should the public sector bear financial risk or act cautiously? And finally, should subsidies be decided only locally, or – following fiscal equivalence – take wider spillover effects into account?

Local authorities can partially mitigate the structural imbalance of bargaining power through measures such as transparent valuation of social and economic benefits, conditional subsidies tied to performance or outcomes, multi-use stadium planning, credible no-bailout commitments, or regional fiscal coordination. These strategies help counterbalance club power and ensure that public interventions achieve broader policy objectives.

While such instruments are useful, they are generally insufficient on their own to guarantee optimal outcomes. The structural characteristics of the football market – including club monopolies, positive externalities, and the political and social significance of clubs – indicate that a more proactive, mission-oriented, and strategically guided approach is necessary. Such questions cannot be settled by economics alone; they require political choices, value judgments, and awareness of the broader context.

### 5.2 Economic policy: from market intervention to strategic investment

Building on the scope conditions and countervailing-power considerations discussed in Section 5.1, it becomes clear that a purely reactive approach to public support is not adequate. Proposition 1 highlights the bilateral monopoly structure and the resulting club market power, while Proposition 2 shows that subsidies can generate social and economic benefits under certain conditions. Together, these findings indicate that local authorities have the opportunity – and the responsibility – to go beyond short-term bailouts and actively shape conditions for sustainable success. A forward-looking approach therefore treats the state as a strategic actor, fostering economic, social, and cultural development rather than merely correcting market failures.

In practice, most public support is reactive, responding to external pressures from clubs, fans, or political actors. Our argument for a strategic, mission-oriented approach builds on this reality, showing how resources can be allocated more efficiently and proactively, with clear rules for when reactive interventions, including crisis support, are appropriate.

Mazzucato's concept of the entrepreneurial state (83) challenges the traditional notion that governments primarily correct market inefficiencies. She argues that the state should act as an entrepreneurial, risk-taking agent that creates public value. She further promotes mission-driven policies (84), where public investment follows clear strategic objectives rather than reacting to crises. Applied to professional football, this means local authorities should not just rescue clubs in crisis but proactively shape conditions for sustainable success – leveraging football as a catalyst for urban development, economic growth, and social cohesion.

Jessop's theory of the entrepreneurial city (85, 86) extends this to urban governance. Cities have evolved from passive administrative bodies to active economic players that strategically position themselves in global markets. In this context, major football clubs can serve as instruments for urban branding, tourism, and economic revitalization. Entrepreneurial cities form strategic partnerships, invest in infrastructure, and host events to stimulate local economies. Accordingly, football subsidies should be seen as integral components of a broader urban strategy to enhance competitiveness, cultural identity, and economic attractiveness.

Reckwitz's concept of embedded liberalism (87) underlines that economic activities, including professional sport, are embedded in cultural and social structures. Modern economies thrive when they integrate cultural capital, symbolic value, and social meaning into their economic strategies. Football clubs are more than commercial enterprises: they represent local identity, community belonging, and symbolic capital. Public investment in football should therefore not be driven solely by financial returns but also strengthen the social and cultural value of clubs within their local context.

Rosentraub (50) calls for a shift from conventional subsidies to strategic, performance-based investments. He criticizes traditional funding models for lacking accountability and long-term impact, instead arguing that public support should be tied to measurable economic and social outcomes. His research demonstrates that when local governments take a strategic, investment-oriented approach, they can transform sport teams into economic drivers rather than financial burdens.

Taken together, these perspectives stress the need for a proactive, mission-oriented approach to local government involvement in professional football. Rather than providing short-term bailouts, authorities should integrate football into wider economic and cultural policies aimed at strengthening urban resilience and identity.

Before engaging with professional football, local governments should address key strategic questions:

- Is the city and region a viable location for professional football, and what league level matches its market potential?
- What initial investment is needed to make the club competitive in line with its market?
- What internal and external benefits are expected, and which strategic goals does public involvement pursue?
- How much ongoing support is required to bridge the gap between market reality and broader policy goals?

On this basis, we advocate a redefined relationship between local authorities and football clubs. The state should act as a strategic sponsor rather than a passive financier, forming structured partnerships with clearly defined goals – a conclusion similarly reached by Hautbois and Desbordes (88). Typical objectives could include:

- City branding and marketing – enhancing global visibility and strengthening the city's image.
- Social and cultural engagement – supporting football's contribution to community development, social inclusion, and cultural identity.
- Talent attraction and retention – making the city more appealing to skilled workers and innovators.
- Business development – using the club as a platform for corporate networking, investment attraction, and economic expansion.

In today's interconnected service economy, networks and value co-creation (6) are crucial. A strategically supported football club can function as a hub for business exchange, community activities, and economic synergy, generating mutual benefits for both the club and the city.

Public engagement should follow the logic of corporate sponsorship: with clear key performance indicators (KPI), focussing on public value, designated budgets for direct support and complementary measures, independent valuation of benefits, clawback provisions, and transparent reporting – ensuring that football-related initiatives are embedded in broader urban strategies and continuously evaluated to optimize economic and social impact.

However, this approach also faces challenges. Measuring brand value and return on investment in sponsorship faces similar methodological difficulties as assessing intangible benefits, with significant uncertainty and potential for lobbying. “Anyway” effects may mean certain benefits would have occurred regardless of public involvement. It is also unclear whether public engagement stimulates (“crowds in”) or discourages (“crowds out”) private sponsorship.

Despite these risks, we argue that a strategic, mission-oriented sponsorship model offers clear advantages. It transforms local authorities from passive supporters into active shapers, balancing market power and benefiting both public stakeholders and clubs alike. Research suggests that ex ante investments are more effective in fostering club development than reactive bailouts (55). Moreover, public funds are allocated more efficiently, reducing the risk of state failure. Clear agreements also help prevent the well-known problem of privatizing gains while socializing losses (83).

### 5.3 Soft power and international relations

A comprehensive analysis of the relationship between professional football and state actors must extend beyond microeconomics to include a geopolitical macro-perspective. Chadwick (89) argues that traditional economic theories fail to capture the complex interplay between sport and global politics. He calls for a geopolitical economy of sport, recognizing that sports are not just economic assets but also instruments of political influence, cultural diplomacy, and international relations. Success in sport is closely linked to success through sport, as nations leverage sporting achievements for geopolitical and competitive advantage (90).

A key mechanism here is soft power, a concept by Nye (91), describing the ability to shape preferences and perceptions through attraction and persuasion rather than coercion or monetary incentives. Nye identifies three primary sources of soft power: culture, political values, and foreign policy.

French scholars Guégan (92) and Boniface (93) have shown that sport has long served as a political tool to project national prestige and exert diplomatic influence. This is visible not only in mega-events like the Olympics but also in European club football. Historically, clubs have embodied state ideologies – most famously Real Madrid, which under Franco's regime symbolized Spanish power through sporting dominance (94).

Today, clubs and leagues are cultural exports and geopolitical tools. Sovereign wealth funds use ownership – like Qatar's PSG, Abu Dhabi's Manchester City, or Saudi Arabia's Newcastle United – to enhance nation branding and soft diplomacy. Sponsorships, such as Rwanda's deal with Bayern Munich or Azerbaijan's with Atlético Madrid, similarly serve image campaigns. State-owned firms like Gazprom or Qatar Airways act as intermediaries integrating football sponsorship into wider foreign policy.

In Germany, the Bundesliga may contribute to national values abroad. The 50 + 1 rule, which reflects democratic principles and community-focused governance, could plausibly function as a geopolitical safeguard against foreign influence, preserving national control over the football sector. We frame this here as an emerging mechanism, noting that further evidence – such as policy documents, parliamentary debates, or DFL/DFB positioning – would be required to substantiate its role. More generally, this dimension of soft power requires long-term planning and mission-oriented policies to be effective.

### 5.4 Limitations and outlook

This study offers a conceptual framework to better understand public subsidies in professional football. By integrating theories from sport economics, microeconomics, strategic management, and geopolitics, it analyzes the complex relationship between clubs and local authorities and highlights key justifications for subsidies. As a conceptual contribution, its primary objective is to provide a structured foundation for future empirical research. This foundation should therefore be expanded through empirical research that reflects the topic's multidisciplinary nature. In particular, future studies could examine how bilateral monopoly structures vary across contexts, including settings with multiple clubs in one location and the role of local substitutes in shaping bargaining dynamics. Moreover, the bargaining position of public authorities acting as payers of last resort deserves closer empirical investigation. Future studies should also draw on new political economy to examine the political drivers and rationales behind state involvement, as well as to provide more robust evidence on opportunity costs, efficiency, and the distribution of benefits.

While our analysis focuses on the German context, particularly the 50 + 1 rule and DFL licensing regulations, the framework can be extended to other European leagues with different ownership and regulatory structures. Future research could examine how variations in investor involvement, governance, and local authority engagement influence the applicability of our propositions and the strategic role of public support. Comparative research drawing on EU state aid practice in sport or alternative stadium financing arrangements could illustrate how governance, investor involvement, and regulatory frameworks shape the strategic role of public support. Such work would enhance the generalizability of our findings across different football systems. Practically, this study encourages policymakers to rethink subsidies, moving from a reactive stance to a strategic and mission-oriented approach.

## Footnotes

^1^The 50 + 1 rule stipulates that a club – or its members – must hold either full or majority ownership (50% plus one vote) of its football team, safeguarding the club from influence by external investors.

^2^Nevertheless, club supporters often influence club managers’ decisions in favor of win maximization (28).

^3^The statutes of the German 3rd league, which is also a professional league and administered by the German Football Association (DFB), are similar in this regard.

^4^18 teams each in first and second Bundesliga, and 20 teams in the third division. Some clubs maintain professional structures in lower divisions, but this is usually highly unprofitable due to limited revenue potential.

^5^The Red Bull corporation accepted this regulatory framework when entering the football market in Leipzig from the grassroots level. However, critics question whether the club's constitution truly meets the requirements of the 50+1 rule.

^6^At the European level, some major clubs from England, Italy, and Spain attempted to break away by proposing an “American style” closed Super League in 2021. However, the project failed due to massive resistance from fans and football associations.

^7^German examples are Bayern Munich and 1860 Munich or Cologne FC and Viktoria Cologne. While there are prominent examples in European football where multiple clubs coexist at a similar level within the same metropolitan area (e.g., Inter and AC Milan, Celtic and Rangers in Glasgow, or several London-based clubs), these cases represent the exception rather than the rule across professional football. An analysis of professional football in the top five European countries (all professional divisions) shows that 81.6% of clubs operate in a local monopoly structure (see appendix).

^8^Madden (2012) distinguishes between three club objectives: profit maximization (P_max_) win maximization (W_max_), and fan welfare maximization (F_max_). In his model, W_max_ clubs deliver the highest sporting quality at the highest prices, P_max_ clubs offer lower quality and attract the smallest audience, while F_max_ clubs reach the largest audience and thus generate the greatest social welfare. This distinction provides an economic justification for the 50+1 rule: by prioritizing fan welfare, total social welfare increases. In our model, we treat win maximization and fan welfare maximization as equivalent.

^9^If the public subsidy is viewed as a reduction of total social value, it is only justifiable up to the point where the difference between TR_S_^P^ and the additional subsidy required to exceed the win-maximizing cost level (TC_S_^P^- TC_C_^W^) is maximized.

## Appendix Table 2 Monopoly clubs in top-5-countries.

**Table:**

Season 2025/26	Germany	France	England	Spain	Italy	Total
Professional football divisions	3	2	4	2	3	14
Clubs (second teams included)	56	36	92	42	100	326
Clubs (second teams excluded)	54	36	92	41	97	320
Locations with several clubs	4	1	8	4	5	22
Non-monopoly clubs	8	3	28	9	11	59
Monopoly clubs	46	33	64	32	86	261
Share monopoly clubs	**85.2%**	**91.7%**	**69.6%**	**78.0%**	**88.7%**	**81.6%**

The final row is highlighted in bold as it reports relative values. While all other rows show absolute figures, the share of monopoly clubs is the key metric, capturing the prevalence of bilateral monopolies and enabling cross-country comparison.
